# *Enterococcus faecium* as an Emerging Pathogen: Molecular Epidemiology and Antimicrobial Resistance in Clinical Strains

**DOI:** 10.3390/pathogens14050483

**Published:** 2025-05-15

**Authors:** Adele Lombardi, Giancarlo Ripabelli, Michela Lucia Sammarco, Manuela Tamburro

**Affiliations:** Department of Medicine and Health Sciences “Vincenzo Tiberio”, University of Molise, 86100 Campobasso, Italy; adelelombardi95@gmail.com (A.L.); sammarco@unimol.it (M.L.S.); manuela.tamburro@unimol.it (M.T.)

**Keywords:** enterococci, multilocus sequence typing, pulsed-field gel electrophoresis, resistance genes, vancomycin resistance, virulence factors

## Abstract

Vancomycin-resistant *Enterococcus faecium* represents an emerging threat in healthcare settings. The aim of this study was to investigate biomolecular characteristics of 31 *E. faecium* isolates from patients in two hospitals of Molise region, central Italy. Particularly, antimicrobial resistance profiles and prevalence of resistance and virulence genes were analyzed, as well as the clonal relationships and sequence types (STs). Antimicrobial susceptibility and genes associated with resistance and virulence were evaluated using automated system and PCR assays, respectively. *SmaI*-based pulsed-field gel electrophoresis (PFGE) and multilocus sequence typing were performed following standardized protocols. All strains exhibited resistance to vancomycin and teicoplanin, and high rates were detected for other antibiotics, except for linezolid. PFGE identified 18 clusters and 26 pulsotypes (Simpson’s index, 0.98). ST80, ST1478, and ST2164 were identified, with ST80 as the most frequent (77.4%). The resistance genes *vanA*, *aac(6*′*)-Ie-aph(2*″*)-Ia*, *aph(3*′*)-IIIa*, and *ermB* were detected in 90.3%, 93.6%, 93.6%, and 90.3% of the strains, respectively, while the *esp* gene was prevalent (61.3%) amongst virulence genes. The study findings highlight the predominance of multidrug-resistant clones and virulence determinants among *E. faecium* strains circulating in the regional hospitals, reinforcing the urgency of implementing targeted molecular surveillance and robust antimicrobial stewardship strategies to contain their spread.

## 1. Introduction

The rise of antimicrobial resistance among vancomycin-resistant enterococci (VRE), particularly in vancomycin-resistant *Enterococcus faecium* (VREfm), has become a critical public health threat worldwide [1]. In recent years, a significant increase in resistance rates in VREfm was reported, primarily driven by high recombination capacity and extensive horizontal gene transfer mechanisms [2,3]. Indeed, the World Health Organization (WHO) has listed VREfm as one of the high-priority pathogens, underlining the urgent need for the development of new antibiotics and the critical importance of effective infection control measures and containment strategies to limit its spread in healthcare settings [4,5].

According to the latest European Centre for Disease Prevention and Control (ECDC) report (2024) [6], *Enterococcus* spp. remains a major contributor to healthcare-associated infections (HAIs) in Europe, with *E. faecium* being the second most isolated species among VRE pathogens in hospital settings, preceded only by *Enterococcus faecalis*. In the 2022−2023 Point Prevalence Survey of HAIs and antimicrobial use in European hospitals, *Enterococcus* spp. was identified in 10.0% of HAI cases, following *Escherichia coli* (12.7%) and *Klebsiella* spp. (11.7%), and more frequently than *Staphylococcus aureus* (9.0%) [6]. Furthermore, VREfm was responsible of 9.6% of HAI cases in Italy (European Centre for Disease Prevention and Control, 2024), contributing to significant morbidity and mortality, particularly among immunocompromised, elderly, and paediatric patients [7,8].

Current data from the Italian National Institute of Health indicate that the overall prevalence of VREfm in invasive infections significantly increased in Europe, with resistance rates towards vancomycin rising from 10.2% in 2015 to 32.5% in 2023 and those related to teicoplanin reaching 31.9% in 2023 [9]. These data highlight the growing difficulty in managing VREfm infections due to their increasing resistance not only to vancomycin but towards other critical antibiotics, including ampicillin (89.7%) and high-dose aminoglycosides (63.5% for streptomycin and 56.6% for gentamicin) [9]. This is of concern because multidrug resistance severely limits treatment options for invasive enterococcal infections, further complicating patient management.

Although vancomycin resistance genes (*vanA*, *vanB*, *vanC*, *vanD*, *vanE*, *vanG*, *vanM*, and *vanL*) have been identified, the most common in VREfm are *vanA* associated with resistance to both vancomycin and teicoplanin, and *vanB* contributing to resistance to vancomycin but not teicoplanin [10]. These predominant genes are carried by mobile genetic elements, such as the transposons Tn1546 and Tn1549/Tn5382, associated with *vanA* and *vanB*, respectively [11].

In addition to vancomycin resistance, VREfm shows significant resistance to β-lactam antibiotics, particularly to ampicillin [12], which is mainly attributed to the overproduction of low-affinity penicillin-binding protein 5 (PBP5) and/or to mutations in the β-subunit of this protein that catalyzes the cross-linking of peptidoglycan during cell wall synthesis [13,14]. Furthermore, the acquisition of genes encoding aminoglycoside-modifying enzymes (AMEs), such as *aph*, *aac*, and *ant*, plays a key role in high-level aminoglycoside resistance [15]. High-level gentamicin resistance in Enterococci is primarily associated with the acquisition of the *aac (6*′*)-Ie-aph (2*″*)-Ia*, *aph (3*′*)-IIIa*, and *ant (3*″*)-III* genes through plasmid-mediated transfer [16]. In addition, *ermB* (erythromycin ribosome methylase), which methylates the bacterial 23S rRNA, is a major contributor to erythromycin resistance [17].

Virulence factors have been also identified in VREfm and are involved in various stages of infection, including aggregation substance (*asa1*) facilitating bacterial adhesion and biofilm formation, gelatinase (*gelE*) degrading extracellular matrix proteins to promote tissue invasion, enterococcal surface protein (*esp*) enhancing colonization and immune evasion, and adhesin to collagen (*ace*) enabling attachment and bacterial dissemination [18].

Since VREfm is one of the most frequently isolated pathogens in nosocomial settings, rapid identification of outbreaks caused by multidrug-resistant (MDR) strains through molecular epidemiological approaches is essential for an effective control [19]. Recent studies emphasize the utility of whole genome sequencing (WGS), providing early detection and enabling prompt response to outbreaks [20]. However, challenges, including data complexity, high costs, and the resolution of highly similar strains, hamper widespread implementation. Nonetheless, molecular methods, such as polymerase chain reaction (PCR) to detect resistance genes, pulsed-field gel electrophoresis (PFGE) for DNA fingerprinting, and multilocus sequence typing (MLST) for evaluating genetic relationships among isolates, continue to play an important role in epidemiological investigations, offering valuable insights into transmission dynamics [19,21].

In this context, the present study aims to characterize *E. faecium* isolated from two hospitals in the Molise region, central Italy, through the investigation of resistance phenotypes to vancomycin, teicoplanin, and other antibiotics; the prevalence of resistance and virulence-associated genes; and clonal relationships between isolates and circulating sequence types (STs).

## 2. Materials and Methods

### 2.1. Strain Selection and Antimicrobial Susceptibility Testing

Thirty-one clinical VREfm isolated between November 2022 and February 2024 were analyzed, with twenty-six (84%) and five (16%) collected from hub and spoke hospitals, respectively.

The isolates were collected through a surveillance system active in the hospital to detect, in a timely manner, the “alert organisms” among hospitalized patients, particularly MDR bacteria, in order to adopt effective containment strategies. The inclusion criteria for strain selection included the isolation from clinical samples collected for diagnostic or surveillance purposes; *E. faecium* species confirmation and antimicrobial susceptibility profile performed by the Microbiology Laboratory of hub hospital during routine diagnostic procedures, using the Phoenix Automated Microbiology System (BD Italy, Milan, Italy) instrument; and non-replicated cultures.

Demographic and clinical data of patients were completely anonymous and were obtained from clinical records and standardized microbiological data sheets. Information included patient age, sex, hospital ward, and sample source, along with laboratory findings such as detailed antimicrobial susceptibility and notes on “*alert organisms*”.

VREfm strains collected at the hospitals were regenerated at the laboratory of the Chair of Hygiene, University of Molise, on Tryptic Soy Agar (Biolife, Milan, Italy) and incubated at 37 °C overnight.

Susceptibility towards vancomycin and teicoplanin was tested for all strains. For nine strains, the following antibiotics were also tested: ampicillin, fusidic acid, clindamycin, cefoxitin, ceftaroline, erythromycin, gentamicin, imipenem, linezolid, and trimethoprim-sulfamethoxazole. Ciprofloxacin was tested for six strains. The minimum inhibitory concentration (MIC) was interpreted according to the latest threshold or breakpoint values established by the European Committee on Antimicrobial Susceptibility Testing [22].

### 2.2. Molecular Typing

Pulsed-field gel electrophoresis (PFGE) of *SmaI*-digested (Promega Corporation, Milan, Italy) bacterial DNA was carried out to examine the relatedness among the isolates, based on the Centers for Disease Control and Prevention (CDC) standardized protocol [23], with modifications of pulse time from 5 to 40 s over 24 h and at 14 °C. Interpretation of restriction patterns was performed by analyzing the dendrogram generated through the UPGMA algorithm and Dice coefficient (BioNumerics software version 5.10, Applied Maths, Sint-Martens-Latem, Belgium).

Furthermore, a validated multilocus sequence typing (MLST) scheme was used [24] to identify allelic profiles and assign the ST. Hence, seven housekeeping genes were amplified by PCR and further sequenced (Eurofins Genomics, Europe Shared Services GmbH, Ebersberg, Germany): *atpA* (ATP synthase alpha subunit), *ddl* (*d*-alanine:*d*-alanine ligase), *gdh* (glucose-6-phosphate dehydrogenase), *purK* (phosphoribosylaminoimidazol carboxylase ATPase subunit), *gyd* (glyceraldehyde-3-phosphate dehydrogenase), *pstS* (phosphate ATP-binding cassette transporter), and *adk* (adenylate kinase). The allelic combination was analyzed on the PubMLST platform (https://pubmlst.org/organisms/enterococcus-faecium, accessed on 5 March 2025).

### 2.3. Detection of Resistance-Associated and Virulence Genes

Total DNA from 31 VREfm strains was extracted using Maxwell^®^ 16 Cell DNA Purification Kit (Promega Corporation, Milan, Italy), according to the manufacturer’s instructions, for the following applications.

The presence of resistance genes was investigated using single PCR assays, including *vanA* and *vanB* (vancomycin resistance) [25], *aac(6*′*)-Ie-aph(2*″*)-Ia*, *aph(3*′*)-IIIa* and *ant(3*″*)-III* (aminoglycoside resistance) [26], *ermB* (macrolide resistance) [27], and *pbp5* (ampicillin resistance) [28]. Through PCRs, the virulence genes *esp* (enterococcus surface protein) [29], *asa1* (aggregation substance) [30], *ace* (adhesion to collagen of enterococcus) [26], and *gelE* (*gelatinase E*) [26] were detected.

Amplifications were performed in a 25 μL final volume with 2.5 μL of DNA template, 1X PCR Master Mix (Promega Corporation), and 0.5–1 μM of each primer. Amplification conditions were as follows: initial denaturation at 94 °C for 3 min, followed by 30 cycles of denaturation (94 °C/1 min), annealing (50 °C/45 s for *vanA*; 55 °C/45 s for *vanB*; 51 °C/1 min for *aac(6*′*)-Ie-aph(2*″*)-Ia*; 54 °C for 1 min for *aph(3*′*)-IIIa*; 53 °C/1 min for *ant(3*″*)-III*, *ermB*; 48 °C/1 min for *pbp5*; 50 °C/1 min for *asa1*; 52 °C/1 min for *ace*; 48 °C/1 min for *gelE*), and extension at 72 °C/1 min, with final extension cycle (72 °C/7 min). Amplified genes were revealed on a 1.0–1.5% agarose gel electrophoresis, using a 100 bp DNA ladder (Promega) and visualized under UV transillumination (UVITEC Cambridge, Fire Reader). Negative and positive controls were included in each PCR assay.

## 3. Results

### 3.1. Characteristics of Strains and Patients and Antimicrobial Resistance Profiles

In this study, clinical *E. faecium* strains were isolated from patients with an average age of 63 ± 22 years (median 69 years; range 0−90) mostly hospitalized in ICUs (n = 22, 70.9%). The isolates were mainly from males (n = 20, 64.5%) and rectal swab (n = 22, 71.0%), followed by urine (n = 4, 12.9%) and blood culture (n = 3, 9.7%) (Table 1).

Overall, all 31 strains tested for teicoplanin and vancomycin were resistant (100%). Furthermore, all nine isolates tested for fusidic acid, clindamycin, cefoxitin, ceftaroline, erythromycin, gentamicin, imipenem, and trimethoprim-sulfamethoxazole exhibited resistance (100%). In addition, seven out of nine (77.8%) and five of six strains (83.3%) tested for ampicillin and ciprofloxacin, respectively, were resistant. Conversely, eight out of nine strains (88.9%) tested for linezolid showed susceptibility.

The *E. faecium* isolates exhibited highly similar multidrug resistance profiles, which were classified into five distinct resistotypes (R1–R5), reflecting consistent patterns of antimicrobial resistance.

The most prevalent was the resistotype R1, accounting for 22 strains (70.9%), all exhibiting resistance to teicoplanin and vancomycin (strains: VREfm 26, 45, 52, 56, 58, 71, 72, 74, 78, 84, 86, 92, 133, 145, 147, 150, 189, 328, 392, 425, 426, 440) and isolated from rectal swab. The resistotype R3 was identified in four strains (16.1%), showing resistance to ampicillin, fusidic acid, ciprofloxacin, clindamycin, cefoxitin, ceftaroline, erythromycin, gentamicin, imipenem, teicoplanin, trimethoprim-sulfamethoxazole, and vancomycin. The strains demonstrated susceptibility to linezolid (strains: VREfm 63, 91, 95, 143) and were isolated from bladder catheter (n = 2, 50.0%), abdominal fluid culture (n = 1, 25.0%), and orotracheal culture (n = 1, 25.0%).

The resistotype R5 was related to three strains (9.7%), with resistance towards ampicillin, fusidic acid, clindamycin, cefoxitin, ceftaroline, erythromycin, gentamicin, imipenem, teicoplanin, trimethoprim-sulfamethoxazole, and vancomycin (strains: VREfm 204, 269, 410), and it was collected from blood culture (n = 2, 66.7%), and bladder catheter (n = 1, 33.3%). The resistotype R4 was linked to one strain (3.2%) from bladder catheter, exhibiting resistance to fusidic acid, ciprofloxacin, clindamycin, cefoxitin, ceftaroline, erythromycin, gentamicin, imipenem, teicoplanin, trimethoprim-sulfamethoxazole, and vancomycin and demonstrating susceptibility to both linezolid and ampicillin (strain: VREfm 348). Similarly, the resistotype R2 was observed in one strain (3.2%) from bladder catheter, displaying resistance to fusidic acid, clindamycin, cefoxitin, ceftaroline, erythromycin, gentamicin, imipenem, linezolid, teicoplanin, trimethoprim-sulfamethoxazole, and vancomycin and susceptibility to ampicillin and ciprofloxacin (strain: VREfm 12).

### 3.2. PFGE Analysis

**Figure 1:**
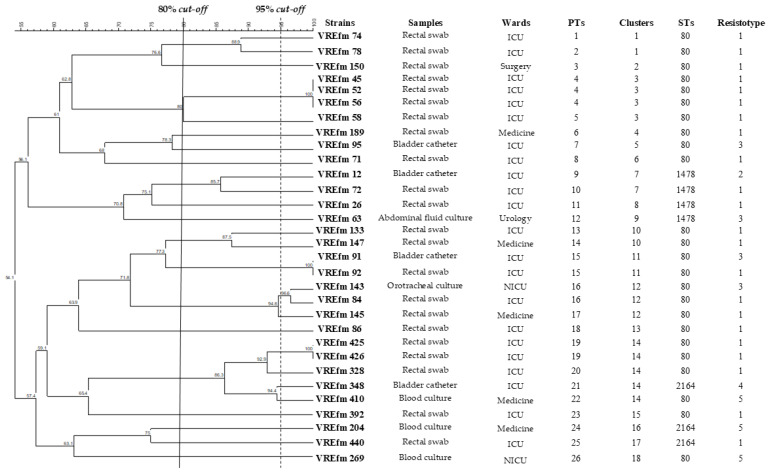
*SmaI*-based dendrogram for 31 *E. faecium* isolates, including type of samples, wards, PTs, clusters, STs, and resistotypes.

PFGE revealed 18 clusters at 80% similarity level (Figure 1), with clusters 14, 3, and 12 as the most prevalent.

Cluster 14 included five strains (VREfm 425, 426, 328, 348, 410) mainly isolated from ICUs (80.0%) in different periods (VREfm 425 and VREfm 426 in February 2024; VREfm 328 and 348 in November 2023; VREfm 410 in January 2024) and mainly isolated from rectal swab (n = 3, 60%) followed by bladder catheter (n = 1; 20%) and blood culture (n = 1, 20%) (Figure 1). Cluster 3 grouped four strains (VREfm 45, 52, 56, 58) all isolated from rectal swab in February 2023 from ICUs and spoke hospitals (50%, respectively). Cluster 12 included three isolates (VREfm 143, 84, 145) from neonatal intensive care units (NICUs), ICUs, and internal medicine departments, reported in the period of March-May 2023 and isolated from rectal swab (n = 2, 66.7%) and orotracheal culture (n = 1, 33.3%).

Twenty-six pulsotypes (PT1-PT26) were observed at 95% cut-off, and Simpson’s index was 0.98. The most prevalent was PT4, related to three strains (VREfm45, 52, and 56), all isolated from the same type of sample (rectal swab) in the same period from ICUs and spoke hospitals. PT15, PT16, and PT19 were each associated with two strains (VREfm 91 and 92; VREfm143 and 84; VREfm425 and 426, respectively) predominantly isolated from rectal swab (n = 4, 66.7%) and from bladder catheter (n = 1) and orotracheal culture (n = 1), (16.7% each) (Figure 1).

### 3.3. Typing by MLST

MLST analysis revealed only three STs (Table 2), and Simpson’s index was 0.39. ST80 with the allelic combination 9-1-1-1-12-1-1 was the most prevalent (n = 24, 77.4%), predominantly isolated from rectal swabs (n = 19, 79.2%), followed by *pstS-null* ST1478 (allelic combination 9-1-1-1-1-0-1) (n = 4, 12.9%), isolated from rectal swab (n = 2, 50.0%%), bladder catheter (n = 1, 25.0%), and abdominal fluid culture (n = 1, 25.0%) and ST2164 (allelic combination 9-141-1-1-12-1-1) (n = 3, 9.68%) amongst strains from bladder catheter (n = 1, 33.3%), rectal swab (n = 1, 33.3%), and blood culture (n = 1, 33.3%). Concerning the isolation time of strains, ST1478 and ST2164 were detected at opposite ends of the isolation period, since ST1478 was identified in isolates from November 2022 to February 2023, while ST2164 emerged in strains from July 2023 to February 2024. In contrast, ST80 was consistently present throughout nearly the entire year analyzed, from February 2023 to February 2024.

Table 3 summarizes the correspondence between STs and the PTs identified for each strain. Notably, ST80 exhibited a high degree of heterogeneity, being distributed across 19 different PTs. In contrast, the less prevalent ST1478 and ST2164 were each associated with four and three distinct PTs, respectively.

### 3.4. Prevalence of Resistance and Virulence-Associated Genes

A total of 28 (90.3%) out of the 31 *E. faecium* strains carried *vanA*, while *vanB* was not detected.

The genes associated with aminoglycoside resistance, including *aac(6*′*)-Ie-aph(2*″*)-Ia* and *aph(3*′*)-IIIa*, were observed in 93.6% of the isolates (n = 29), and *ermB* was present in 90.3% (n = 28). None of the strains harbored *ant(3*″*)-III* or *pbp5*.

Amongst virulence genes, *esp* was identified in a high proportion of isolates (61.3%, n = 19), while *asa1*, *ace*, and *gelE* genes were found in one strain (VREfm 91).

## 4. Discussion

The present study highlights the prevalence of VREfm strains in hospitalized patients in the Molise region, central Italy, providing valuable insights into molecular characteristics and antimicrobial resistance patterns.

The phenotypic profile of the strains strongly correlated with clinical risk factors, particularly patient age and ICU admission, as reported in a recent study [31]. A significant proportion (65%) of isolates were from elderly patients, a demographic population group often associated with increased susceptibility to infections due to underlying health conditions and prolonged hospitalization [32]. Additionally, most isolates were from ICUs, where the combination of invasive procedures, antibiotic pressure, and critical patient conditions create a favorable environment for the emergence and dissemination of MDR pathogens [33].

Of note, the predominance of rectal swabs as the primary sample source suggests that gastrointestinal colonization must be accurately evaluated, being critical for the dissemination of *E. faecium* [34]. This finding underscores the need for rigorous screening to prevent the spread of resistant strains, particularly in ICUs where vulnerable patients are at greater risk [35].

The resistance to vancomycin and teicoplanin observed in all isolates is consistent with data from the Antimicrobial Resistance Surveillance Network from the Italian Institute of Health [9], highlighting a high prevalence of vancomycin and teicoplanin resistance, 32.5% and 31.9% respectively, among *E. faecium* isolates in Italy during 2023. Considering the most recent European data, Italy ranked among the countries with the highest resistance rates (40.0%) for *E. faecium* in Europe, following Greece (72.2%) and Lithuania (73.3%). This underscores the urgent need for robust antimicrobial stewardship programs in Italian healthcare facilities.

The resistance was mainly mediated by the *vanA* gene, although the possible involvement of alternative mechanisms could be related to VREfm isolates lacking this gene. These may include the presence of genes in the *van* cluster other than *vanB*, including *vanD*, *vanE*, or *vanG*, which confer glycopeptide resistance [36]; mutations in genes involved in cell wall synthesis and remodeling (*ddl*, *murF*) [37]; as well as intrinsic mechanisms of resistance mediated by efflux pumps [38]. These findings highlight the need for comprehensive genomic analyses to fully elucidate the role of these possible pathways.

Our result is concordant with previous evidence [39], reporting one of the highest prevalence rates of the *vanA* gene among vancomycin resistance determinants. Compared to other resistance-associated genes—such as *vanB*, *vanD*, *vanE*, and *vanG—vanA* stands out as the dominant mechanism, further reinforcing its key role in vancomycin resistance in *E. faecium.*

Indeed, the *vanA* gene cluster, often located on the transposon Tn1546, can spread both clonally and horizontally through plasmid dissemination or transposition between genomic locations [40].

In contrast, the *vanB* gene was not observed in our study, as supported by previous reports from Italian hospitals, reporting only sporadic detection of *vanB*-positive strains [41,42], compared to other European countries [43].

The resistance found in all isolates towards fusidanes (fusidic acid), lincosamides (clindamycin), β-lactams (cefoxitin, ceftaroline), macrolides (erythromycin), aminoglycosides (gentamicin), carbapenems (imipenem), and sulfonamides-trimethoprim (trimethoprim-sulfamethoxazole) highlights the multidrug-resistant phenotype which limits effective treatment options. This alarming resistance profile is consistent with a previous study [44] reporting high resistance rates among *E. faecium* isolates in Italy, with 86.7% resistant to imipenem. Similarly, significant resistance patterns were observed in *E. faecium* clinical isolates, especially against ciprofloxacin (70.9%), followed by high-level resistance against gentamicin (39.4%) [45]. The resistance rates for ampicillin (77.8%) and ciprofloxacin (83.3%) are similarly alarming, as these antibiotics are often among the first-line treatments for *E. faecium* infections [46]. Ampicillin resistance is strongly associated with alterations in the *pbp5* gene [47], although our findings did not identify this gene, suggesting that this resistance is probably mediated by alternative mechanisms or genetic determinants. Potential contributing factors include mutations in the *pbp5* gene that alter the structural affinity for β-lactams [48] or mutations in the cell wall synthesis regulatory genes, which can reduce β-lactam affinity, or increased expression of β-lactamases that degrade ampicillin [49].

Further resistance determinants were widely prevalent among the isolates, with *aac(6*′*)Ie-aph(2*″*)-Ia* and *aph(3*′*)-IIIa* genes reflecting aminoglycoside resistance. The *ermB* gene, responsible for macrolide resistance, was also commonly detected, corroborating the distinct genetic profile exhibited by these isolates.

The susceptibility to linezolid in most strains offers a viable therapeutic alternative although the emergence of linezolid-resistant *E. faecium* has already been reported in other settings [50].

Concerning the virulence factors, a significant proportion of strains carried the *esp* gene, which has been associated with enhanced colonization and persistence in hospital settings [37]. This result was consistent with other findings reporting high prevalence of this gene in clinical *E. faecium* isolates and related to biofilm formation, persistence, and transmission in healthcare settings [51].

In contrast, the lower prevalence of *asa1*, *ace*, and *gelE* was consistent with previous evidence showing that these genes are more commonly associated with *Enterococcus faecalis* than *E. faecium* [52]. Indeed, the pathogenicity in *E. faecium* mainly depends on the presence of the *esp* gene, which is known to play a key role in biofilm formation and colonization of host tissues [53].

To investigate *E. faecium* epidemiology, PFGE and MLST were used in this study, as they are still the preferred methods. PFGE analysis revealed distinct cluster-specific patterns, with cluster 14 being predominantly associated with an ICU environment. In detail, three strains (VREfm 425, 426, and 328) were isolated from rectal swabs, one (VREfm 348) from bladder catheter, and one (VREfm 410) from blood culture. This emphasizes the critical role that ICUs play as focal points for the transmission of VREfm, typically harboring patients with compromised immune systems or subjected to a high frequency of invasive medical procedures. Moreover, this clustering raises concerns about environmental reservoirs, such as equipment or healthcare workers’ contamination [54]. This necessitates an infection prevention approach including regular decontamination of surfaces, enhanced training for healthcare staff, and the utilization of advanced diagnostic tools for early detection and intervention [55].

However, VREfm isolates were isolated from multiple wards, including Internal Medicine, NICU, Urology, and General Surgery. Notably, cluster-specific patterns observed in these wards reveal important epidemiological insights and underline the risk posed by VREfm in non-ICU settings. For example, cluster 12, including the isolates from ICUs and internal medicine departments, suggests potential cross-ward transmission events or shared sources of contamination. This underlines the need for targeted infection control measures [56].

Further epidemiological insights emerged from cluster 3, which grouped four isolates (VREfm 45, 52, 56, 58), all recovered in February 2023 from ICUs and spoke hospital (50% each). Although the temporal and spatial clustering might suggest intra-hospital transmission, these isolates were obtained from rectal swabs collected as part of hospital admission screening.

Nonetheless, PFGE analysis revealed that three out of four isolates within cluster 3 were clonally related, reinforcing the hypothesis of a common transmission source. The persistence of clonally identical strains across different locations in the same period suggests that VREfm spread could have been facilitated by reservoirs within the hospital environment, implying a focused transmission event.

In contrast, the isolation dates of the strains included in cluster 14 cover a broad period (from November 2023 to February 2024), suggesting a persistent presence of these strains within the ICU. However, many isolates were from rectal swabs performed at admission; thus, patients were colonized.

Furthermore, the high genetic diversity observed across VREfm isolates (Simpson’s Index, 0.98) and the presence of 26 distinct pulsotypes highlights the complexity of transmission dynamics, including patient transfers, contaminated medical equipment, and transient healthcare reservoirs.

In addition, the considerable genetic variability across isolates suggests that VREfm is undergoing continuous adaptation to selective pressures within the hospital environment. This variation may be driven by intensive antibiotic use, favoring resistant subpopulations and accelerating the genetic diversification of strains through mutations and horizontal gene transfer.

Concerning MLST, ST80 was the predominant ST, consistently identified amongst strains from various wards. This finding was concordant with other Italian studies [57,58], which also highlighted the association of ST80 with clonal complex CC17, known for its role in HAIs and the ability to harbor resistance determinants, thus supporting its significance in transmission dynamics.

The distinct temporal clustering of ST1478 and ST2164 appearing at opposite ends of the isolation period suggests possible episodic introductions or localized outbreaks. These patterns may warrant further epidemiological investigation to identify potential transmission sources, assess the role of hospital or environmental reservoirs, and determine whether specific antimicrobial resistance mechanisms contributed to their emergence.

Conversely, the presence of ST80 throughout the study period suggests its sustained circulation within the hospital environment, potentially indicating endemic persistence or ongoing transmission dynamics that require continued monitoring. Similar observations were reported in a recent study [59], identifying ST1478 as a *pstS-null* ST in Italian hospitals. This sequence type was associated with vancomycin-variable *E. faecium* isolates, suggesting a unique genetic adaptation that may contribute to its sporadic emergence. The identification of ST2164 is also in line with findings from other Italian regions, where this ST has been linked to specific outbreaks in acute care settings [59].

As observed in Table 3, strains with identical PTs were consistently assigned to the same STs. For example, PT4 was related to three strains, all with ST80, while PT15, PT16, and PT19 were each linked to two isolates with ST80. Notably, ST80 was the predominant ST and represented by a wide range of PTs (PT1–PT8, PT13–PT20, PT22–PT23, PT26).

In contrast, ST1478 and ST2164 were associated with a restricted set of pulsotypes. Indeed, ST1478 corresponded to four PTs (PT9–PT12), while ST2164 corresponded to three (PT21, PT24, PT25). The presence of these STs suggests the emergence of distinct clonal types.

The clusters identified through PFGE showed high concordance with MLST results, because most of them were composed exclusively of isolates belonging to a single ST. This consistency suggests that both methods reliably capture the clonal structure of *E. faecium* populations. For instance, the largest PFGE cluster included several PTs all assigned to ST80, while others were composed of strains belonging to either ST1478 or ST2164, with no evidence of mixed STs within the same PFGE cluster.

Overall, findings indicate that these typing methods provide complementary insights into the molecular epidemiology of *E. faecium*. While PFGE offers a high-resolution approach to detect genetic differences between strains, MLST provides a more stable and reproducible method for identifying major clonal lineages. This is further supported by the Simpson’s diversity indices, with PFGE showing higher discriminatory power (0.98) compared to MLST (0.39), reflecting its ability to distinguish closely related isolates within the same lineage. Indeed, both PFGE and MLST are reliable tools for tracking the spread and evolution of MDR pathogens in the hospital environment.

These correlations underscore the importance of continuous molecular surveillance to monitor the dynamics of predominant and emerging STs [60], enabling timely interventions to prevent the spread of resistant *E. faecium* strains [61].

The prevalence of strains in non-ICU settings also highlights the potential role of inter-ward patient transfers and shared healthcare equipment in facilitating transmission [62]. By addressing both ICU and non-ICU sources of transmission, a more comprehensive approach to infection control can be achieved, ultimately reducing the burden of VREfm in healthcare facilities.

This study has limitations, including the relatively small sample size and the focus on a single geographical area which may not fully reflect the broader epidemiological trends. Further research should explore the characteristics of a higher number of strains and include genomic analyses to better understand the resistance mechanisms and transmission pathways.

Based on the study findings, active surveillance programs should be reinforced, especially in high-risk wards such as ICUs, integrating genomic surveillance and environmental sampling to elucidate the role of possible reservoirs and transmission routes and adopting strict infection control measures, such as contact precautions and antimicrobial stewardship, to prevent the spread of VREfm and that of other MDR pathogens.

## 5. Conclusions

*E. faecium*, with its multifaceted resistance profiles and virulence traits, represents a significant challenge in hospital settings, particularly in ICUs where patients are highly vulnerable to nosocomial infections. This study demonstrated that resistance to vancomycin is highly related to the *vanA* gene, while *vanB* strains were irrelevant. Information achieved through the classical methodologies of PFGE and MLST facilitated the identification of clusters and the characterization of circulating STs linked to clones. However, the integration in future investigations with WGS-based data could offer more detailed insights into the genetic landscape and transmission dynamics of these strains.

Despite the relatively small number of isolates tested, the study findings are significant not only at local level, as could be important in contributing to the description of the epidemiological framework. Moreover, as the study is related to an area previously unexplored in the context of VREfm prevalence and molecular characterization, it provides a valuable foundation for future research, offering insights that could inform effective interventions and contribute to the advancement of regional and national healthcare strategies.

## Figures and Tables

**Table 1:** List of *E. faecium* strains included in the study, with demographic characteristics (age, sex) of patients and clinical data (ward, hospital type, sample source, and collection date between November 2022 and February 2024).

Strains	Ward	Hospital	Age	Sex	Sample	Collection Date
VREfm 12	ICU	Hub	70	M	Bladder catheter	November 2022
VREfm 26	ICU	Hub	53	M	Rectal swab	November 2022
VREfm 45	ICU	Hub	82	M	Rectal swab	February 2023
VREfm 52	ICU	Spoke	78	F	Rectal swab	February 2023
VREfm 56	ICU	Hub	49	M	Rectal swab	February 2023
VREfm 58	ICU	Spoke	69	F	Rectal swab	February 2023
VREfm 63	Urology	Hub	59	M	Abdominal fluid culture	February 2023
VREfm 71	ICU	Spoke	90	M	Rectal swab	February 2023
VREfm 72	ICU	Hub	67	M	Rectal swab	February 2023
VREfm 74	ICU	Spoke	74	M	Rectal swab	February 2023
VREfm 78	ICU	Hub	41	F	Rectal swab	March 2023
VREfm 84	ICU	Hub	75	F	Rectal swab	March 2023
VREfm 86	ICU	Hub	53	M	Rectal swab	March 2023
VREfm 91	ICU	Hub	41	F	Bladder catheter	March 2023
VREfm 92	ICU	Spoke	64	M	Rectal swab	March 2023
VREfm 95	ICU	Hub	41	F	Bladder catheter	March 2023
VREfm 133	ICU	Hub	80	M	Rectal swab	April 2023
VREfm 143	NICU	Hub	1	F	Orotracheal culture	May 2023
VREfm 145	Medicine	Hub	82	M	Rectal swab	May 2023
VREfm 147	Medicine	Hub	72	M	Rectal swab	May 2023
VREfm 150	Surgery	Hub	74	M	Rectal swab	May 2023
VREfm 189	Medicine	Hub	49	M	Rectal swab	June 2023
VREfm 204	Medicine	Hub	79	F	Blood culture	July 2023
VREfm 269	NICU	Hub	0	F	Blood culture	August 2023
VREfm 328	ICU	Hub	67	M	Rectal swab	November 2023
VREfm 348	ICU	Hub	69	M	Bladder catheter	November 2023
VREfm 392	ICU	Hub	69	M	Rectal swab	December 2023
VREfm 410	Medicine	Hub	89	F	Blood culture	January 2024
VREfm 425	ICU	Hub	83	M	Rectal swab	February 2024
VREfm 426	ICU	Hub	83	F	Rectal swab	February 2024
VREfm 440	ICU	Hub	83	M	Rectal swab	February 2024

**Table 2:** Allelic designation for *atpA*, *ddl*, *gdh*, *purK*, *gyd*, *pstS*, and *adk* genes in the MLST scheme and sequence type (ST) identification among 31 *E. faecium* strains, including sample type and ward.

Strains	Sample	Ward	*atpA* Allele	*ddl* Allele	*gdh* Allele	*Purk* Allele	*gyd* Allele	*pstS* Allele	*adk* Allele	ST
VREfm 12	Bladder catheter	ICU	9	1	1	1	1	0	1	1478
VREfm 26	Rectal swab	ICU	9	1	1	1	1	0	1	1478
VREfm 45	Rectal swab	ICU	9	1	1	1	12	1	1	80
VREfm 52	Rectal swab	ICU	9	1	1	1	12	1	1	80
VREfm 56	Rectal swab	ICU	9	1	1	1	12	1	1	80
VREfm 58	Rectal swab	ICU	9	1	1	1	12	1	1	80
VREfm 63	Abdominal fluid culture	Urology	9	1	1	1	1	0	1	1478
VREfm 71	Rectal swab	ICU	9	1	1	1	12	1	1	80
VREfm 72	Rectal swab	ICU	9	1	1	1	1	0	1	1478
VREfm 74	Rectal swab	ICU	9	1	1	1	12	1	1	80
VREfm 78	Rectal swab	ICU	9	1	1	1	12	1	1	80
VREfm 84	Rectal swab	ICU	9	1	1	1	12	1	1	80
VREfm 86	Rectal swab	ICU	9	1	1	1	12	1	1	80
VREfm 91	Bladder catheter	ICU	9	1	1	1	12	1	1	80
VREfm 92	Rectal swab	ICU	9	1	1	1	12	1	1	80
VREfm 95	Bladder catheter	ICU	9	1	1	1	12	1	1	80
VREfm 133	Rectal swab	ICU	9	1	1	1	12	1	1	80
VREfm 143	Orotracheal culture	NICU	9	1	1	1	12	1	1	80
VREfm 145	Rectal swab	Medicine	9	1	1	1	12	1	1	80
VREfm 147	Rectal swab	Medicine	9	1	1	1	12	1	1	80
VREfm 150	Rectal swab	Surgery	9	1	1	1	12	1	1	80
VREfm 189	Rectal swab	Medicine	9	1	1	1	12	1	1	80
VREfm 204	Blood culture	Medicine	9	141	1	1	12	1	1	2164
VREfm 269	Blood culture	NICU	9	1	1	1	12	1	1	80
VREfm 328	Rectal swab	ICU	9	1	1	1	12	1	1	80
VREfm 348	Bladder catheter	ICU	9	141	1	1	12	1	1	2164
3.3VREfm 392	Rectal swab	ICU	9	1	1	1	12	1	1	80
VREfm 410	Blood culture	Medicine	9	1	1	1	12	1	1	80
VREfm 425	Rectal swab	ICU	9	1	1	1	12	1	1	80
VREfm 426	Rectal swab	ICU	9	1	1	1	12	1	1	80
VREfm 440	Rectal swab	ICU	9	141	1	1	12	1	1	2164

**Table 3:** Correspondence between sequence types (STs) and pulsotypes (PTs) among 31 *Enterococcus faecium* isolates, including the number of strains belonging to each PT.

STs	PTs	Strains	N. of Strains
ST80 (identified for 24 strains)	1	VREfm 74	1
	2	VREfm 78	1
	3	VREfm 150	1
	4	VREfm 45, VREfm 52, VREfm 56	3
	5	VREfm 58	1
	6	VREfm 189	1
	7	VREfm 95	1
	8	VREfm 71	1
	13	VREfm 133	1
	14	VREfm 147	1
	15	VREfm 91, VREfm 92	2
	16	VREfm 143, VREfm 84	2
	17	VREfm 145	1
	18	VREfm 86	1
	19	VREfm 425, VREfm 426	2
	20	VREfm 328	1
	22	VREfm 410	1
	23	VREfm 392	1
	26	VREfm 269	1
ST1478 (identified for 4 strains)	9	VREfm 12	1
	10	VREfm 72	1
	11	VREfm 26	1
	12	VREfm 63	1
ST2164 (identified for 3 strains)	21	VREfm 348	1
	24	VREfm 204	1
	25	VREfm 440	1

## Data Availability

This study includes all data, although the authors are available for further explanations. This was a non-interventional study, and the main findings were related to strains collected within the “alert organisms” surveillance system established in the hospital; hence, for patient data, anonymity was assured. Since this study focused on the molecular characterization of bacterial isolates, the research did not involve humans, and ethical approval from an ethics committee was not required.

## Abbreviations

The following abbreviations are used in this manuscript:

VRE	Vancomycin-resistant enterococci
VREfm	Vancomycin-resistant *Enterococcus faecium*
WHO	World Health Organization
HAIs	Healthcare-associated infections
ECDC	European Centre for Disease Prevention and Control
ICU	Intensive care unit
TSA	Tryptic Soy Agar
PFGE	Pulsed-field gel electrophoresis
CDC	Centers for Disease Control and Prevention
UPGMA	Unweighted pair group method with arithmetic mean
MLST	Multilocus sequence typing
PCR	Polymerase chain reaction
PT	Pulsotype
MDR	Multidrug resistant
NICU	Neonatal intensive care unit
CC	Clonal complex
